# Entry of Oncolytic Herpes Simplex Virus into Human Squamous Cell Carcinoma Cells by Ultrasound

**DOI:** 10.3390/v7102890

**Published:** 2015-10-26

**Authors:** Shusuke Okunaga, Ayako Takasu, Noritoshi Meshii, Tomoaki Imai, Masakagu Hamada, Soichi Iwai, Yoshiaki Yura

**Affiliations:** 1 Department of Oral and Maxillofacial Surgery, Osaka University Graduate School of Dentistry, 1-8 Yamadaoka, Suita, Osaka 565-0871, Japan; migakedentan@mail.goo.ne.jp (S.O.); a-takasu@dent.osaka-u.ac.jp (A.T.); tomoaki@dent.osaka-u.ac.jp (T.I.); hmdmskz@dent.osaka-u.ac.jp (M.H.); s-iwai@dent.osaka-u.ac.jp (S.I.); 2 Dental and Oral Surgery, Izumisano Municipal Hospital, 2-23 Rinkuoraikita, Izumisano, Osaka 598-8577, Japan; ich_liebe_dich_auch@msn.com

**Keywords:** oncolytic HSV-1, squamous cell carcinoma, ultrasound, microbubble

## Abstract

Low-intensity ultrasound is a useful method to introduce materials into cells due to the transient formation of micropores, called sonoporations, on the cell membrane. Whether oncolytic herpes simplex virus type 1 (HSV-1) can be introduced into oral squamous cell carcinoma (SCC) cells through membrane pores remains undetermined. Human SCC cell line SAS and oncolytic HSV-1 RH2, which was deficient in the γ_1_34.5 gene and fusogenic, were used. Cells were exposed to ultrasound in the presence or absence of microbubbles. The increase of virus entry was estimated by plaque numbers. Viral infection was hardly established without the adsorption step, but plaque number was increased by the exposure of HSV-1-inoculated cells to ultrasound. Plaque number was also increased even if SAS cells were exposed to ultrasound and inoculated with RH2 without the adsorption step. This effect was abolished when the interval from ultrasound exposure to virus inoculation was prolonged. Scanning electron microscopy revealed depressed spots on the cell surface after exposure to ultrasound. These results suggest that oncolytic HSV-1 RH2 can be introduced into SAS cells through ultrasound-mediated pores of the cell membrane that are resealed after an interval.

## 1. Introduction

Oncolytic virotherapy with herpes simplex virus type-1 (HSV-1) is now under clinical application for the treatment of cancer because the results obtained for the efficiency of oncolytic HSV-1 in advanced malignant melanoma and head and neck cancer in phase I/II studies are promising [1,2,3]. The most reliable method by which to deliver oncolytic HSV-1 to solid tumors is direct inoculation. After being injected into tumors, oncolytic HSV-1 must spread in the microenvironment of tumors and reach its susceptible cells. One important characteristic of the tumor microenvironment is the combination of leaky vasculature and lack of functional lymphatics, which can create increased interstitial fluid pressures [4,5] and it is not easy for inoculated viruses and progeny viruses to spread throughout tumor tissue. The efficient entry of the inoculated virus into tumor cells may be required to achieve a significant antitumor effect by oncolytic HSV-1.

Low-intensity ultrasound does not damage irradiated cells, but transiently produces pores on the cell membrane and allows extracellular materials including DNA, chemicals, and viruses to enter cells [6,7]. This pore-producing effect of ultrasound, called sonoporation, was shown to be elevated in the presence of microbubbles, which were initially used as a contrast agent for ultrasound diagnosis [8,9]. We reported that exposure to ultrasound could increase the efficiency of HSV-1 infection if cells were exposed to ultrasound after an incubation of cells with virus for 30 min [10,11], indicating that ultrasound promotes the entry of HSV-1 when it was applied after an incubation of virus with susceptible cells to facilitate virus binding to their receptors. Regarding pore formation by ultrasound, it is evident that pores are acting as sieves, allowing molecules to pass if their size is below the pore diameter. Adeno-associated virus (AAV) has been used as a vector to carry genomic DNAs into cells through the pore-producing effect of ultrasound [12,13], however, its ability to facilitate the entry of oncolytic HSV-1 – which is larger than AAV – through pores remains unclarified. In the present study, we investigated whether oncolytic HSV-1 RH2 [14,15] that was deficient in the γ_1_34.5 gene, a neurovirulent gene responsible for encephalitis and exhibiting fusogenic ability, enters into human oral squamous cell carcinoma (SCC) cells thorough ultrasound-mediated membrane pores.

## 2. Materials and Methods

### 2.1. Cells

The human oral SCC cell line SAS was obtained from the Japanese Collection of Research Bioresources (Tokyo, Japan). SAS cells were cultured in Dulbecco’s modified Eagle’s medium supplemented with 10% fetal bovine serum, 2 mM l-glutamine, 100 U/mL penicillin, and 100 μg/mL streptomycin and grown in an incubator at 37 °C in a humidified atmosphere with 5% CO_2_.

### 2.2. Viral Infection

HSV-1 RH2, which exhibits a fusogenic ability in human SCC cells, is deficient for the γ_1_34.5 gene and has mutations in glycoprotein B [14,15], was grown in semi-confluent Vero cell monolayers. Infected cells were subjected to three cycles of freezing and thawing and were then centrifuged at 3000 × *g* for 15 min at 4 °C. The supernatant was kept at -80 °C prior to use. Vero cell monolayers were infected with the virus serially diluted 10–fold for a standard plaque assay to determine the virus titer. Cells were inoculated with the virus and incubated at 37 °C for 60 min to ensure virus binding to their receptors. Thereafter, unbound viruses were removed by washing cell monolayers with phosphate-buffered saline (PBS), which were covered with a medium containing 0.3% methylcellulose. They were incubated at 37 °C in a humidified atmosphere with 5% CO_2_ for approximately 48 h. After the development of cytopathic changes, plaques were counted and plaque-forming unit (PFU)s/mL was determined. Plaque formation in SAS cells was also performed in a similar manner as described in Vero cells.

### 2.3. Ultrasound Exposure

The Artison microbubble (Artison Corp, Inola, OK, USA) is a lipid-shelled ultrasound contrast agent filled with perfluorocarbon gas; it is composed of 5 × 10^8^ microbubbles/mL, with an average diameter of 2.4 μm [16]. An ultrasound machine, Sonitron 2000V (Nepagene Japan, Chiba, Japan), was used. For ultrasound irradiation after HSV-1 inoculation, cells were grown on alternate 24-well polystyrene plates (Corning, NY, USA) to prevent exposure to neighboring cells [17]. Microbubbles were mixed with the virus, after which 100 μL of a mixture containing 5 × 10^7^ microbubbles was added to the cell cultures. Cells were exposed to ultrasound in the presence or absence of microbubbles at room temperature. The transducer was firmly fixed to a stand to avoid dislocation during the exposure to ultrasound, the plates were placed on the head of the transducer with a diameter of 12 mm, and contact was mediated using an ultrasound contact gel. The ultrasound frequency was 1 MHz throughout the experiments. Ultrasound was adjusted to supply an intensity of 1.0 W/cm^2^ at a duty cycle of 20%, based on the findings of previous studies [10,11]. In the case of 96-well plate, a transducer with a diameter of 8 mm was used.

### 2.4. Plaque Formation in Combination with Ultrasound

**Figure 1:**
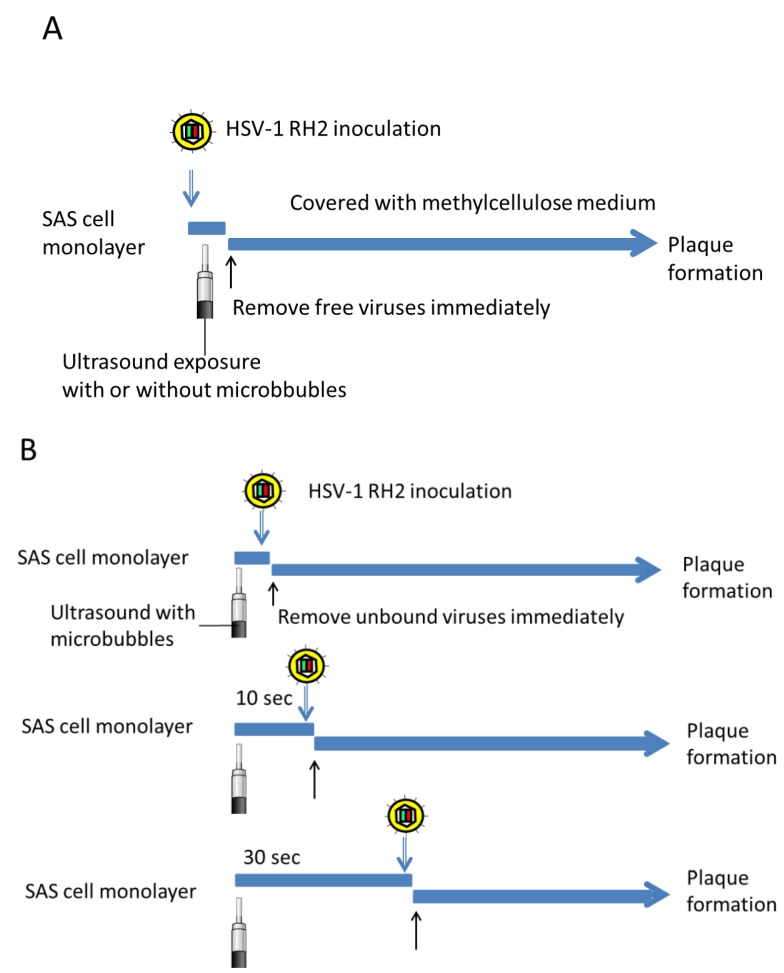
Experiments to determine the ultrasound-mediated increase of virus entry estimated by plaque numbers. (**A**) SAS cell monolayers were inoculated with HSV-1 RH2, with or without microbubbles, and then exposed to ultrasound for 10 s at room temperature. Cell monolayers were immediately washed with PBS to remove any unbound virus, covered with medium containing 0.3% methylcellulose and cultured at 37 °C for the plaque assay; (**B**) SAS cells were treated with ultrasound for 10 s in the presence of microbubbles at room temperature. At 10 to 1200 s after ultrasound exposure, cells were inoculated with HSV-1 RH2. They were immediately washed with PBS to remove unbound virus and covered with medium containing 0.3% methylcellulose for the plaque assay.

For the study of ultrasound exposure immediately after viral inoculation, SAS cell monolayers were inoculated with 1 × 10^4^ PFU of HSV-1 RH2, with or without 5 × 10^7^ microbubbles, and then exposed to ultrasound for 10 s at room temperature. Cell monolayers were immediately washed with PBS to remove any unbound virus, covered with medium containing 0.3% methylcellulose and cultured at 37 °C for the plaque assay (Figure 1A). Thus, inoculated viruses were washed out from the cell cultures within 30 s to minimize adsorption step of HSV-1.

To determine the interval during which virus entry was permitted without an adsorption time, cell monolayers were treated with ultrasound in the presence of microbubbles at room temperature. After 10 to 1200 s, cells were inoculated with 5 × 10^3^ PFU of HSV-1 RH2. They were immediately washed with PBS to remove unbound viruses prior to being covered with medium containing 0.3% methylcellulose for the plaque assay (Figure 1B).

### 2.5. 3-(4,5-Dimethylthiazol-2-yl)-2,5-Diphenyl-Tetrazolium Bromide (MTT) Assay

Cells were grown on alternate 96-well plates. Ten microliters of a 5 mg/mL MTT (Sigma, St. Louis, MO, USA) solution was added to each well with 100 μL of medium, and cells were incubated at 37 °C for 4 h. After the addition of 100 μL of 0.04N HCl in isopropanol, the plates were mixed thoroughly to dissolve the dark blue crystal and left to stand at room temperature overnight. The plates were read on a Benchmark Plus microplate spectrophotometer (Bio-Rad Laboratories, Hercules, CA, USA) with a reference wavelength of 630 nm and a test wavelength of 570 nm. Background absorbance at 630 nm was subtracted from the 570 nm reading. The values were divided by those of the control and the rate was calculated.

### 2.6. Scanning Electron Microscopy

Treated cells were fixed with 2.5% glutaraldehyde in PBS for 1 h. The fixed cells were rinsed with PBS several times, then dehydrated in a series of graded concentrations of ethanol, and finally substituted with tert-butyl alcohol. The specimens were freeze-dried, sputter-coated with platinum, and observed with a scanning electron microscope (SEM: S-4300; Hitachi Co., Tokyo, Japan).

### 2.7. Statistical Analysis

Results are reported as means ± standard deviation (SD). An Anova test was used to determine the significance of differences in multiple comparisons. Comparisons between each treatment group and the control were achieved by Student’s *t*-test. These statistical analyses were performed using the software Statcel3 (OMS, Tokyo, Japan). A value of *p* < 0.05 was considered to be significant.

## 3. Results

### 3.1. Effect of Ultrasound on the Growth of Oral SCC Cells

**Figure 2:**
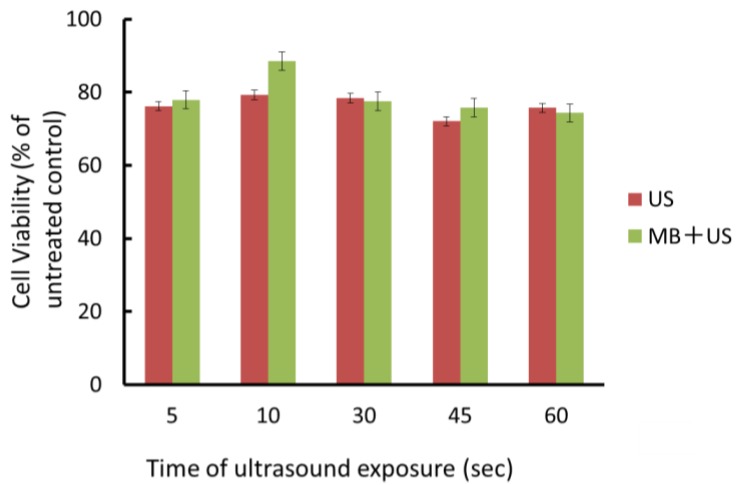
Effects of ultrasound on the viability of oral SCC cells. SAS cells were plated on 96-well plates and grown. They were exposed to ultrasound at an intensity of 1 W/cm^2^, and 20% duty cycle in the presence or absence of microbubbles at room temperature for various periods of time and then incubated at 37 °C for 48 h. The viability of treated cells was examined by the MTT assay. Data are means (± SD) of six determinations. US: ultrasound; MB: microbubble.

Whether ultrasound in the presence or absence of microbubbles could affect the growth of SAS was examined at an intensity of 1 W/cm^2^ and 20% duty cycle for 10–60 s. Cells were exposed to ultrasound and viability was evaluated using the MTT assay. Cell viability at 1 W/cm^2^ was approximately 80% of the untreated control during an exposure time of 60 s. Even if microbubbles were added to the medium and exposed to ultrasound, a suppressive effect on cell viability was not observed (Figure 2). The results described above indicated that microbbubles ultrasound only did not decrease cell viability.

### 3.2. Effects of Ultrasound Exposure Immediately after Viral Inoculation on HSV-1 Plaque Formation and Cell Viability

**Figure 3:**
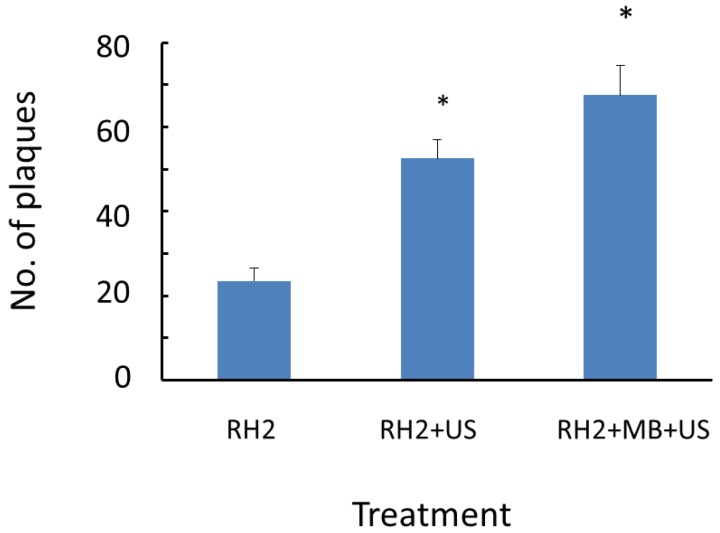
Effects of ultrasound immediately after the viral inoculation on HSV-1 plaque formation. SAS cells were inoculated with 1 × 10^4^ PFU of HSV-1 RH2 with or without microbubbles. Thereafter, cells were exposed to ultrasound at an intensity of 1 W/cm^2^, and 20% duty cycle for 10 s. Cell monolayers were immediately washed with PBS to remove unbound virus and covered with medium containing methyl cellulose. For plaque assay, these cells were cultured at 37 °C for approximately 48 h. Results are means (± SD) of four determinations. * *p* < 0.05 significantly different from control (RH2).

Whether viral infection could occur in the absence of adsorption step was examined. SAS cells were inoculated with HSV-1 RH2 and then exposed to ultrasound for 10 s. Thereafter, unbound viruses were immediately removed by washing with PBS, covered with medium containing methyl cellulose and incubated for approximately 48 h until plaque formation (Figure 1A). Only 22 plaques (0.2%) were produced in control cultures that received 1 × 10^4^ PFU of HSV-1 RH2, but this number was increased 3-fold when ultrasound was combined with microbubbles (Figure 3).

### 3.3. Effects of the Interval from Ultrasound Exposure to Viral Inoculation on HSV-1 Plaque Formation

**Figure 4:**
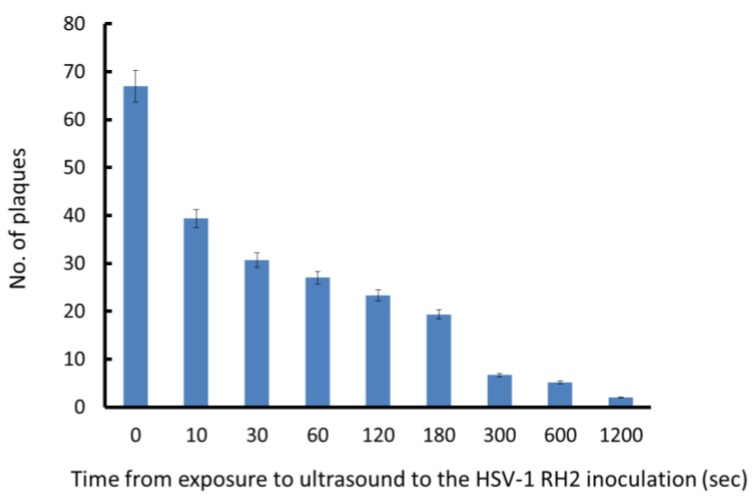
Effect of ultrasound exposure and delayed viral inoculation on HSV-1 RH2 plaque formation. SAS cells were exposed to ultrasound at first. After incubation at room temperature for 10 to 1200 s, cells were inoculated with 5 × 10^3^ PFU of HSV-1 RH2 and then immediately washed with PBS to remove unbound virus and plaque formation was performed at 37 °C. Results are means (± SD) of three determinations.

If HSV-1 RH2 uses temporal pores created in cell membrane by ultrasound as a route of entry, ultrasound exposure prior to virus inoculation may also be effective to increase the entry of virus. To examine this possibility, SAS cells were firstly exposed to ultrasound with microbubbles for 10 s. After 10 to 1200 s, cells were inoculated with HSV-1 RH2 and unbound viruses were immediately removed (Figure 1B). The plaque number was maximal when cells were infected immediately after exposure to ultrasound, and decreased when the time between ultrasound and viral inoculation was prolonged (Figure 4). The plaque number decreased to the untreated control level 1200 s after exposure to ultrasound.

### 3.4. Scanning Electron Microscope of Oral SCC Cells Exposed to Ultrasound

**Figure 5:**
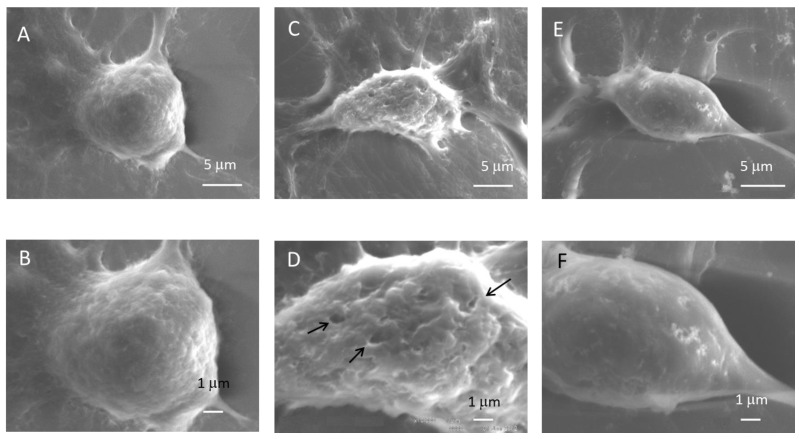
Scanning electron microscopy of oral SCC cells incubated with HSV-1 and treated with ultrasound. SAS cells grown on cover glass were processed for scanning electron microscopy (**A**,**B**) or exposed to ultrasound in the presence of microbubbles at room temperature (**C**,**D**); the cells were exposed to ultrasound and then incubated for 30 min at 37 °C prior to be scanned by electron microscopy (**E**,**F**). Arrows indicate depressions on the cell surface.

When untreated SAS cells were examined with scanning electron microscopy, they were spherical in shape, with a diameter of approximately 10 μm. The cell surface had a number of small protrusions and extending processes were observed at the cell periphery. Immediately after exposure to ultrasound in the presence of microbubbles, depressed spots of 0.4–0.6 μm were observed on the cell surface (Figure 5). Most cell processes and spots were lost and the cell surface became smooth 30 min after exposure to ultrasound.

## 4. Discussion

### 4.1. Increase of Virus Entry by Ultrasound in the Presence or Absence of Microbubbles

The inoculated HSV-1 may bind to cellular receptors and enter into cells through membrane fusion or endocytosis during the period of adsorption. At least three of the 12 types of glycoproteins of the envelope bind to cell surface receptors leading to the entry of HSV-1 into cells. Initially, glycoprotein gB and gC bind to heparan sulfate proteoglycans on the cell surface, attaching the virus to the host cell. Once the viral and host cell membranes are close to each other, glycoprotein gD can associate with any of a number of cell receptors, including the herpes virus entry mediator, nectin-1, and 3-*O*-sulfated heparin sulfate, and triggers fusion [18]. Depending on the cell line, HSV-1 can enter by direct fusion of the viral envelope with the plasma membrane or by endocytic pathways [19]. We previously examined the effects of ultrasound on plaque formation after an adsorption time of 30 min and found that plaques were significantly higher following exposure to ultrasound than in the control condition. Since all of the inoculated viruses did not initiate a replication cycle during the incubation for 30 min, ultrasound may have promoted the entry of viruses that bind to the cell surface. In this situation, the virus entry estimated by plaque number was increased and 40-50% of inoculated virus entered the cells [11].

It has been suggested that cavitation plays a pivotal role in ultrasound-mediated gene transfer. Mechanical shear stress, microstreaming or some other type of energies generated by cavitation could affect plasma membrane forming transient pores. Microbubbles, which provide cavitation nuclei, significantly enhance ultrasound-mediated gene transfer [6]. Geers *et al.* [20] reported on the ultrasound-assisted delivery of non-enveloped AAV particles with diameters of 20 nm, and showed that AAV was directly delivered into the cytosol of cells by sonoporation. To examine the possibility that enveloped HSV-1 particles with diameters of 100–150 nm could be also introduced into cells through pores, experiments were undertaken in the absence of the sufficient binding time of viruses to their cellular receptors. We chose the ultrasound condition to be 1 W/cm^2^, 20% duty cycle, for 10 s [10,11]. Microbubbles were used at the non-cytotoxic concentration (Figure 2).

Plaque formation was minimal in control cultures in which unbound viruses were removed by washing immediately after the inoculation. However, the plaque number was increased three-fold by exposure to ultrasound in presence of microbubbles; therefore the increase was attributed to the virus entering cells through pores in the plasma membrane. To further examine this possibility, we performed ultrasound exposure first and then cells were inoculated with HSV-1. We found that plaque formation occurred even if the order of viral inoculation and ultrasound was exchanged.

If viruses utilize pores to enter cells, the pores will be resealed after an interval; therefore, the amounts of the virus that enter cells in the absence of an adsorption step will be reduced. As expected, the plaque number was decreased by prolonging the interval between exposure to ultrasound and virus inoculation. In the present study, the enhancing effect was lost after 20 min. Our scanning electron microscopy study showed that the pores on cell membranes were lost after 30 min (Figure 5). Mehier-Humbert *et al.* [21] examined the effects of ultrasound on the entry of carboxylate-modified nanoparticles to reduce non-specific binding with the plasma membrane, which is also negatively-charged by sonoporation, and found that the percentage of particles that entered was reduced to 4.3% after 5 min. Together, these results support the conclusion that HSV-1 can be introduced into cells through transient pores on the cell membrane: how introduced HSV-1 starts its replication cycle remains unclear.

### 4.2. Scanning Electron Microscope of Oral SCC Cells

A previous scanning electron microscopy study showed that the pore upper-size limit was close to 75 nm, though pore size distribution was likely to be heterogenous [21]. Zhou *et al.* [22] reported that the mean radius of pore size was determined as 110 nm. Maeda *et al.* [23] indicated that the size of pores produced by cancer cells was 2 μm. In the present study, we examined cells immediately after exposure to ultrasound and detected depressed spots on the cell surface, especially after exposure to ultrasound with microbubbles. The size was considered to be 0.4–0.6 μm, being sufficient for HSV-1 with the average size of 100–150 nm to pass. It is not known whether the observed depressed spots represent pores on the cell membrane caused by ultrasound.

Recently, we demonstrated that ultrasound exposure to the HSV-1-injected tumor increased the expression of viral antigen and enhanced the antitumor ability of the oncolytic virus [11], indicating that this method may be useful for ensuring the infection of oncolytic HSV-1 at the site of inoculation, and subsequent oncolysis due to the replication of HSV-1. In the present study, the increase of plaque number due to pore-mediated entry of HSV-1 by sonoporation was approximately 0.4% of the inoculated virus; the amounts were much lower than those introduced by receptor- mediated entry of HSV-1. HSV-1-injected tumors should be exposed to ultrasound after an incubation time of virus with tumor cells or exposed repeatedly to ensure the entry of virus by receptor-mediated entry as well as pore-mediated entry of HSV-1.

## 5. Conclusions

Whether injected viruses can initiate their infection cycles successfully depends on the adsorption step in which viruses can associate with tumor cells. Nevertheless, ultrasound may facilitate the entry of HSV-1 into SCC cells through transient pores of the cell membrane without the adsorption step.
